# Identification and Profiling of MicroRNAs in the Embryonic Breast Muscle of Pekin Duck

**DOI:** 10.1371/journal.pone.0086150

**Published:** 2014-01-23

**Authors:** Lihong Gu, Tieshan Xu, Wei Huang, Ming Xie, Shiduo Sun, Shuisheng Hou

**Affiliations:** 1 Shaanxi Key Laboratory of Molecular Biology for Agriculture, College of Animal Science and Technology, Northwest A&F University, Yangling, Shaanxi, P.R. China; 2 Institute of Animal Science (IAS), Chinese Academy of Agricultural Sciences (CAAS), Beijing, P.R. China; CSIR-Central Drug Research Institute, India

## Abstract

MicroRNAs (miRNAs) regulate gene expression by fully or partially binding to complementary sequences and play important roles in skeletal muscle development. However, the roles of miRNAs in embryonic breast muscle of duck are unclear. In this study, we analyzed the miRNAs profiling in embryonic breast muscle of Pekin duck at E13 (the 13^th^ day of hatching), E19, and E27 by high-throughput sequencing. A total of 382 miRNAs including 359 preciously identified miRNAs 23 novel miRNA candidates were obtained. The nucleotide bias analysis of identified miRNAs showed that the miRNAs in Pekin duck was high conserved. The expression of identified miRNAs were significantly different between E13 and E19 as well as between E27 and E19. Fifteen identified miRNAs validated using stem-loop qRT-PCR can be divided into three groups: those with peak expression at E19, those with minimal expression at E19, and those with continuous increase from E11 to E27. Considering that E19 is the fastest growth stage of embryonic Pekin duck breast muscle, these three groups of miRNAs might be the potential promoters, the potential inhibitors, and the potential sustainer for breast muscle growth. Among the 23 novel miRNAs, novel-miRNA-8 and novel-miRNA-14 had maximal expression at some stages. The stem-loop qRT-PCR analysis of the two novel miRNAs and their two targets (MAP2K1 and PPARα) showed that the expression of novel-mir-8 and PPARα reached the lowest points at E19, while that of novel-mir-14 and MAP2K1 peaked at E19, suggesting novel-miRNA-8 and novel-miRNA-14 may be a potential inhibitor and a potential promoter for embryonic breast muscle development of duck. In summary, these results not only provided an overall insight into the miRNAs landscape in embryonic breast muscle of duck, but also a basis for the further investigation of the miRNAs roles in duck skeletal muscle development.

## Introduction

In birds, a primary and a secondary generation of fibers arise respectively during the embryonic and fetal stages of development. Following these two waves of myogenesis, the total number of fibers is fixed [1] and there are no significant changes in fiber numbers during later bird development [2], [3]. Birds, such as poultry, are therefore attractive models for studying muscle development during the prenatal development and has become a research focus [4]. The family of myogenic regulatory factors (MRFs), which includes MyoD, Myf5, MRF4, and MyoG, is important for embryonic muscle development [5]. The members of MRFs coordinate the expression of genes involved in muscle growth, morphogenesis, muscle cell differentiation and contractility. Recently, it has been shown that miRNAs play important roles in skeletal muscle development [6], [7].

MicroRNAs (miRNAs) are short (approximately 22 nucleotides) noncoding RNA molecules that bind to complementary mRNAs sequences, hereby promoting mRNA degradation or translational repression [8]–[10]. An essential role of miRNAs in skeletal muscle development is evidenced since the deletion of Dicer which is responsible for the maturation of miRNAs results in perinatal lethality due to skeletal muscle hypoplasia [11]. In particular, the critical roles of three muscle-specific miRNAs, miR-1, miR-133 and miR-206, in the regulation of myogenesis have been well documented [6], [12] with miR-1 and miR-133 regulating different aspects of skeletal muscle development both *in vitro* and *in vivo* [13]. The miR-1 promotes muscle cell differentiation by repressing the expression of histone deacetylase 4 (HDAC4), an inhibitor of muscle differentiation. In C2C12 myoblasts, miR-133a promotes proliferation by partially repressing serum response factor (SRF). Like miR-1, miR-206 promotes differentiation of C2C12 myoblasts *in vitro* by repressing the expression of the DNA polymerase A subunit (Polal) [14], connexin 43 (Cx43) [15], follistatin-like 1 (Fstl1) and utrophin (Utrn) [16]. In addition, other miRNAs have also been shown to play a role in muscle development. Over expression of miR-181 during muscle cell differentiation is important in promoting myogenesis by down-regulating the homeobox protein Hox-A11, an inhibitor of myogenesis [17]. The miR-486 has been shown to induce myoblast differentiation by down-regulating Pax7 [18], while miR-27b regulates Pax3 translation and ensures myogenic differentiation [19]. Recently, studies have shown that miR-148a positively regulates myogenic differentiation via down-regulating Rho-associated coiled-coil containing protein kinase 1 (ROCK1), a known inhibitor of myogenesis and miR-214 may target the negative regulators of Myf5, MyoD and myogenin in the corresponding stages of skeletal muscle development in vivo to regulate embryonic myogenesis [5]. It has recently been evidenced that miRNAs is one of the most abundant players of gene regulatory molecules in vertebrates.

Currently, there are approximate 21264 predicted hairpin miRNAs and 25141 novel mature miRNAs from 193 species in the publicly available miRNA database miRBase (Release 19.0, August 2012) (http://www.mirbase.org). It is surprising that there is no duck miRNAs presented in the miRBase because duck not only has tremendous agricultural importance [20]–[22] but also is a natural reservoir of influenza A viruses [23], [24]. A few studies have begun to explore duck miRNAs in various aspects. Zhang et al. profiled miRNAs in duck feather follicle and skin with high-throughput sequencing technology [25]. Powder et al. identified and compared the miRNAs expressed in cranial NC cells from three avian species (chicken, duck, and quail) before and after species-specific facial distinctions occur [26]. In addition, the novel and differentially expressed miRNAs in the ovaries of laying and non-laying duck have been identified by Yu [27]. However, the analysis of miRNAs in many other tissues, including skeletal muscle, is still deficient. Therefore, exploring of miRNAs in duck skeletal muscle will greatly improve the understanding of the role of miRNAs in avian skeletal development.

In a preliminary study we observed that the growth rate of embryonic breast muscle of Pekin duck reached its peak at the 19^th^ day of hatching (E19) and the expression level of muscle regulatory factor 4 (MRF4), coincidentally, peaked at E19 (unpublished data). These results indicated that E19 is the fastest growth stage of embryonic breast muscle of Pekin duck, yet the underlying molecular mechanism regulating this rapid growth stage is still unclear. Given the important roles of miRNAs in skeletal muscle development, identification of the differentially expressed miRNAs in different developmental stages is a critical first step to investigate the function of miRNAs in embryonic muscle development in ducks.

Here, we analyzed miRNA expression from embryonic breast muscle of Pekin duck at developing stage of E13 (the 13^th^ day of hatching), E19, and E27 by high throughput sequencing. With bioinformatics analysis and stem-loop qRT-PCR validation of some identified and novel miRNAs, we identified differentially expressed miRNAs in embryonic breast muscles in duck and hereby providing a basis for further investigation on the molecular mechanisms of breast muscle development in duck.

## Results and Discussion

### High-throughput sequencing and small RNA discovery

**Figure 1:**
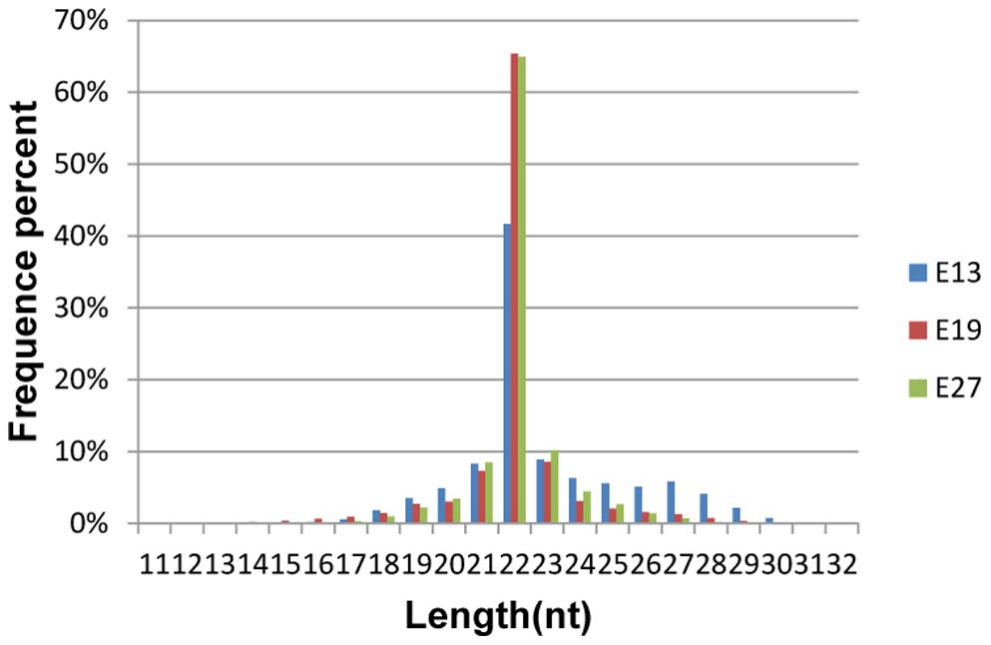
Length distribution of sequence reads after quality trimming and adaptor removal. Note: E13, E19 and E27 represent the breast muscle libraries of the 13^th^, 19^th^, and 27^th^ day of hatching.

**Figure 2:**
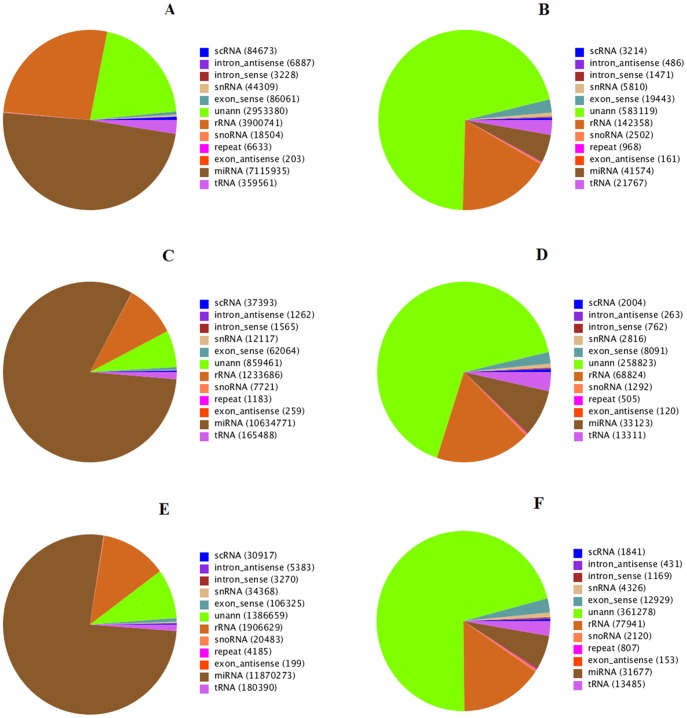
Pie charts distribution of the annotation of assigned small RNAs at stage E13, E19 and E27. Note: Total small RNAs of E13 (A), Unique small RNAs of E13 (B), Total small RNAs of E19 (C), Unique small RNAs of E19 (D), Total small RNAs of E27 (E) and Unique small RNAs of E27 (F).

Total RNAs from embryonic breast muscle of Pekin duck at stage E13, E19 and E27 were used to construct small RNA libraries for high throughput sequencing. A total of 14881453, 13411560 and 15775148 raw reads were obtained from the E13, E19 and E27 libraries, respectively. After quality control and adaptor removal, 14580115, 13016970 and 15549081 high-quality reads were available from E13, E19 and E27 libraries, respectively (Table 1) for further analysis. Length distribution analysis showed that most reads ranged from 21–23 nt with the percentage of the 22 nt reads of the total reads being 41.63%, 65.34% and 64.92% for the three libraries, respectively (Fig. 1). The high-quality reads were subsequently annotated to different classes of RNA categories (identified miRNAs, repeats-associated RNA, rRNA, tRNA, snRNA, snoRNA, etc) using different databases such as miRBase (V19.0), from Rfam(10.1) and Genbank (Table 2, Fig. 2). The most abundant RNA species (based on read count) in the three libraries was classified as miRNAs, accounting for 48.41%, 81.70% and 76.34% in the three libraries, respectively. This indicates that the deep sequencing data were highly enriched for mature miRNA sequences and that the data are well suitable for expression profiling analysis of identified miRNAs and discovering of novel miRNAs. The second most abundant category was rRNAs, accounting for 26.75%, 9.48% and 12.26% in the three libraries, respectively. In addition, unknown RNAs also represented a high percentage (20.26%, 6.60% and 12.26%, respectively). Finally, all reads were aligned against the chicken genome (http://hgdownload.cse.ucsc.edu/goldenPath/galGal3/bigZips/chromFa.tar.gz) using the program SOAP 2.0 [28]. A total of 10273938, 11171826 and 13010462 reads from E13, E19 and E27 libraries, respectively, aligned perfectly to the chicken genome (Table S1).

**Table 1:** Summary of small RNA sequencing data.

Type	Count-E13	%-E13	Count-E19	%-E19	Count-27	%-E27
**Total reads**	14881453		13411560		15775148	
**High quality**	14794223	100%	13376800	100%	15691433	100%
**3′adaptor null**	1986	0.01%	6332	0.05%	1824	0.01%
**Insert null**	2620	0.02%	1613	0.01%	2281	0.01%
**5′adaptor contaminants**	69355	0.47%	38647	0.29%	67052	0.43%
**Small than18 nt**	139922	0.95%	313055	2.34%	71069	0.45%
**PolyA**	225	0.00%	183	0.00%	126	0.00%
**High quality reads**	14580115	98.55%	13016970	97.31%	15549081	99.09%

**Table 2:** Distribution of the genome-mapped sequence reads in small RNA libraries.

Locus class	Uni-sRNA-E13	Tot-sRNA-E13	Uni-sRNA-E19	Tot-sRNA-E19	Uni-sRNA-E27	Tot-sRNA-E27
**Total reads**	822873	14580115	389934	13016970	508157	15549081
**Exon antisense**	161(0.02%)	203(0.00%)	120(0.03%)	259(0.00%)	153(0.03%)	199(0.00%)
**Exon sense**	19443(2.36%)	86061(0.596%)	8091(2.07%)	62064(0.48%)	12929(2.54%)	106325(0.68%)
**Intron antisense**	486(0.06%)	6887(0.05%)	263(0.07%)	1262(0.01%)	431(0.08%)	5383(0.03%)
**Intron sense**	1471(0.18%)	3228(0.02%)	762(0.20%)	1565(0.01%)	1169(0.23%)	3270(0.02%)
**miRNA**	41574(5.05%)	7115935(48.41%)	33123(8.49%)	10634771(81.70%)	31677(6.23%)	11870273(76.34%)
**rRNA**	142358(17.30%)	3900741(26.75%)	68824(17.65%)	1233686(9.48%)	77941(15.34%)	1906629(12.26%)
**Repeat**	968(0.12%)	6633(0.05%)	505(0.13%)	1183(0.01%)	807(0.16%)	4185(0.03%)
**scRNA**	3214(.039%)	84673(0.58%)	2004(0.51%)	37393(0.29%)	1841(0.36%)	30917(0.20%)
**snRNA**	5810(0.71%)	44309(0.30%)	2816(0.72%)	12117(0.09%)	4326(0.85%)	34368(0.22%)
**snoRNA**	2502(0.30%)	18504(0.13%)	1292(0.33%)	7721(0.06%)	2120(0.42%)	20483(0.13%)
**tRNA**	21767(2.65%)	359561(2.47%)	13311(3.41%)	165488(1.27%)	13485(2.65%)	180390(1.16%)
**Unannotated reads**	583119(70.86%)	2953380(20.26%)	258823(66.38%)	859461(6.60%)	361278(71.10%)	1386659(8.92%)

### Identification and evaluation of identified miRNAs

Currently, there is no miRNAs database of duck in the miRBase. We therefore aligned our small RNA reads against the miRNA precursor/mature miRNA of all animals in the miRBase (V19.0) and extracted the sequence and count of miRNA families (no specific species) which were presented in our samples. The criterions for identifying identified miRNAs in the breast muscle tissues of Pekin duck were as follows: Firstly, to account for the difference among species, we aligned the reads to the precursor/mature miRNAs of all animals in miRBase allowing two mismatches and free gaps. Secondly, the highest expressed miRNA for each mature miRNA family was used to construct a miRNA database which was used to align the reads to estimate the expressions of the miRNAs by counting the number of reads aligning to each miRNA in the database. Thirdly, we predicted the precursor of the identified miRNAs and discarded the miRNAs without a predicted hairpin structure.

**Figure 3:**
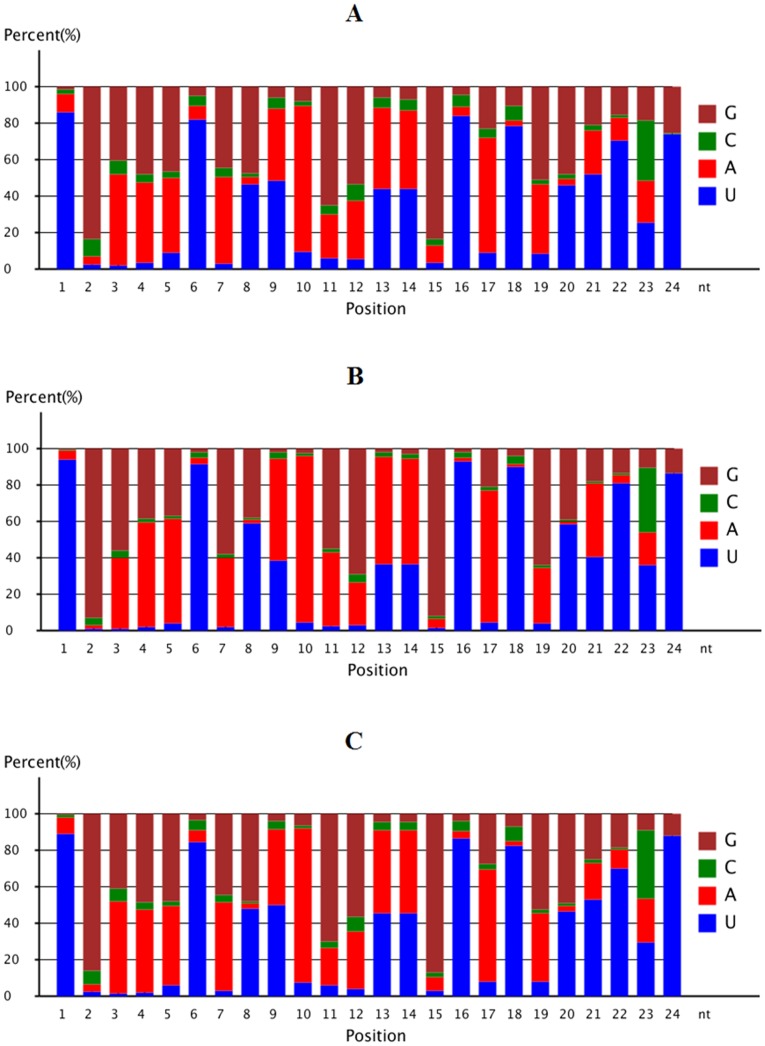
miRNA Nucleotide distribution at each position. Note: E13 (A), E19 (B) and E27 (C).

Among the identified miRNAs, we first investigated their base preference. It has been suggested that the bases of 1^st^ and 9^th^ position from 5′ terminal which are responsible for targeting mRNAs for gene regulation, and the 3′ terminal positions are enriched with a U base [29]. In the current study, we obtained similar results from E13, E19 and E27 stages. From the three stages, U accounted for 85.9%, 93.9% and 88.7%, respectively at the 1^st^ position, 48.2%, 38.4% and 50.0%, respectively for the 9st position and 74.0%, 86.2% and 87.6% respectively, for the 5′ terminal (Fig. 3, Table S2). The bases preference of duck miRNAs are consistent with the statistical results through previous large-scale genome analysis performed by Zhang [30] and indicated that miRNAs in duck, like in other species, is highly conserved.

A total of 359 mature miRNAs, belonging to 262 families, were identified from the E13, E19 and E27 libraries (Table S3). The largest miRNA family identified was Let-7 consisting 21 members. Many other families, such as miR-1, miR-10, miR-1013, miR-124, miR-128, miR-130, miR-133, miR-206 and miR-148 contained two members, whereas most identified families had only one member. The finding that many members of identified miRNA families in all other animals were expressed in the breast muscle of Pekin duck suggested that a broad scale miRNAs are needed for muscle development of duck. Different family members also displayed drastically different expression levels. For example, the abundance of the Let-7 family varied from 3 read (let-7a-2-3p) to 2149081 reads (let-7f-5p). This was also the case for some other miRNA families, such as miR-499 (from 1to 10316 reads), miR-199 (from 1004 to 256996 reads) and miR-10 (from 17 to 6058 reads). The existences of a dominant member in a miRNA family suggest that the regulatory role of certain family is performed by the dominant member at a specific stage.

After literature mining, 24 of the identified miRNAs were involved in skeletal muscle development (Table 3). Almost all of these muscle-specific miRNAs (myomiRs) were highly expressed. The most abundant one was miR-1a-3p (Table S3), which was represented by 1906033, 5142560, and 2847317 reads in E13, E19 and E27 libraries, respectively (Table 3) which is in line with the distinct role of miR-1 in modulating skeletal muscle cell proliferation and differentiation [13]. miR-206 and miR-206-3p (4^th^ and 5^th^ abundance, Table S3) accounted for more than 760,000 reads in each library, which is consistent with their established function in skeletal muscle development [52] and reported roles in myogenesis [53], [54]. Compared with the above myomiRs, different types of miR-133 were expressed at relatively low levels in the skeletal muscle libraries: there were 3715, 4912 and 7043 reads for miR-133, and 2791,4253 and 5151 reads for miR-133-3p in the three libraries, respectively (Table S3). These variations in global expression of muscle-specific miRNAs could reflect different roles of these miRNAs in myogenesis [13], [38]. In addition, miR-107, miR-103a-3p, miR-128, miR-222a, miR-26a-5p, miR-221, miR-146b-5p, miR-16c, and miR-130a, which have been implicated as the muscle-related miRNAs, also showed high count number (Table 3). The fact that muscle-specific or muscle-related miRNAs are highly enriched indicated that the data extracted from the duck breast muscle are credible and that the expression patterns of miRNAs identified in this study likely reflect the expression characteristics of miRNAs in the three muscle tissues.

**Table 3:** miRNAs identified both in the embryonic breast muscle of Pekin duck and identified to be muscle-related from literatures.

miR-name	Sequence	Cou-E13	Cou-E19	Cou-E27	Studies related to muscle
**miR-1**	TGGAATGTAAAGAAGTATGTAG	24092	173545	36785	[13], [30]
**miR-103a-3p**	AGCAGCATTGTACAGGGCTATGA	81126	56571	162616	[13]
**miR-107**	AGCAGCATTGTACAGGGCTAT	103914	71695	208096	[31]
**miR-10a-5p**	CAAATTCGTATCTAGGGGAAT	22	17	57	[32]
**miR-125b-5p**	TCCCTGAGACCCTAACTTGTGA	7565	4471	12624	[33]
**miR-128**	TCACAGTGAACCGGTCTCTTTT	55580	45584	106122	[34]
**miR-130a**	CAGTGCAATATTAAAAGGGCAT	11450	6118	21728	[13]
**miR-133**	TTTGGTCCCCTTCAACCAGCT	3715	4912	7043	[13], [30]
**miR-146b-5p**	TGAGAACTGAATTCCATAGGCGTT	5902	12899	13048	[35], [36]
**miR-148a**	TCAGTGCACTACAGAACT	2663	2888	4138	[5]
**miR-16c**	TAGCAGCACGTAAATACTGGAG	10156	11154	20798	[37]
**miR-181b**	AACATTCATTGCTGTCGGTGGGTT	67868	30759	95543	[38]
**miR-1a-3p**	UGGAAUGUAAAGAAGUAUGUAU	1906033	5142560	2847317	[13], [30]
**miR-206**	TGGAATGTAAGGAAGTGTGTG	769238	1129210	1799320	[39]–[41]
**miR-20b-3p**	ACTGTAATGTGGGCACTTA	63	226	95	[13]
**miR-21-3p**	AACAACAGTCGGTAGGCTGTCT	495	112	754	[37], [42]
**miR-214**	TACAGCAGGCACAGACAG	3273	2997	5955	[43], [44]
**miR-221**	AGCTACATTGTCTGCTGGGTTT	15833	13285	32726	[45], [46]
**miR-222a**	AGCTACATCTGGCTACTGGGTCT	7451	25999	15333	[45], [46]
**miR-23a**	ATCACATTGCCAGGGATTTCCA	4008	5680	7679	[47]
**miR-24**	TGGCTCAGTTCAGCAGGAACAGT	3542	5477	7708	[48]
**miR-26a-5p**	TTCAAGTAATCCAGGATAGGCT	14031	22315	24219	[49]
**miR-29b**	TAGCACCATTTGAAATCAGTGT	4	19	27	[50]
**miR-99a-5p**	AACCCGTAGATCCGATCTTGTG	3673	2312	6471	[13], [51]

**Figure 4:**
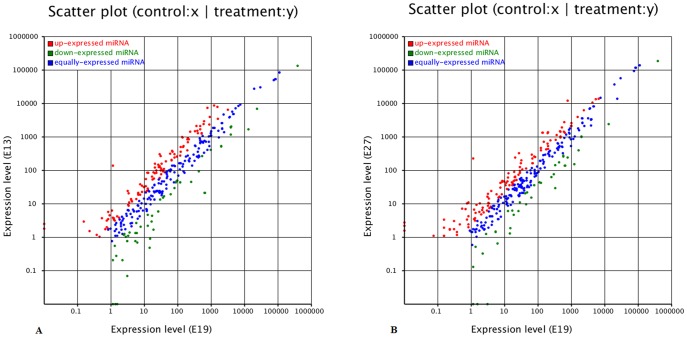
Differentially expressed miRNAs between stages. Note: (1) E13 and E19 (A), and stage E19 and E27 (B). (2) Each point represents a miRNA. Red points represent miRNAs with a fold change >2, blue points represent miRNAs with 1/2<a fold change <2, green points represent miRNAs with a fold change ≤1/2.

The main objective of the present study is to identify the miRNAs which are involved in the regulation of embryonic breast muscle development in duck. The expression of identified miRNAs in the three samples was assessed by plotting Log2-ratio and Scatter Plot (Fig. 4). The expression profiles among the three libraries are shown in Table S4. Between E19 and E13 libraries a total of 333 differentially expressed at a higher level miRNAs were observed, of which 207 miRNAs genes were expressed in E13 than in E19 and 126 miRNAs genes were expressed at a lower level in E13 than in E19. Among these differentially expressed miRNAs, 160 miRNAs were extremely significantly different and 6 were significantly different and the fold-change (log2 E13/E19) ranged between −7.26 (miR-202-5p) and 7.95 (miR-2995). For E19 and E27 libraries there were 342 differentially expressed miRNAs, of which 240 were higher expressed in E27 compared to E19 and 102 were lower expressed in E27 compared to E19. A total of 161 miRNAs were extremely significantly different and 3 were significantly different and the fold-change (log2 E27/E19) ranged from −8.23 (miR-6087) to 8.08 (miR-2995) among the differentially expressed miRNAs. Combining the results of differentially expressed miRNAs among the three libraries and the fact that E19 is the stage with fastest growth in embryonic breast muscle development of duck, the miRNAs with significantly different expression at stage E19 compared to stages E13 and E27 may play important roles in fine-tuning the required gene expression for fast growth of muscle.

### Prediction of novel miRNA

The characteristic hairpin structure of a miRNA precursor can be used to predict novel miRNA. The Mireap software [http://sourceforge.net/projects/mireap/] was used to predict novel miRNAs by exploring the secondary structure, the Dicer cleavage site and the minimum free energy of the small unassigned high-quality reads. The following key conditions are used as criteria for assigning novel miRNAs: (1) the small unassigned high-quality reads must map to an intron region or an antisense exon region of a reference genome; (2) Sequence structures must satisfied the following two criteria: hairpin miRNAs can fold secondary structures and mature miRNAs are present in one arm of the hairpin precursors; (3) The mature miRNA strand and its complementary strand have 2-nucleotide 3′ overhangs; (4) Hairpin precursors lack large internal loops or bulges; (5) The secondary structures of the hairpins are steady, with a free energy of hybridization lower than or equal to −18 kcal/mol.

Based on the criteria described above, 23 (11, 5 and 18 from E13, E19 and E27, respectively) novel miRNA candidates were obtained (Table 4). Because genomic information of duck are not available, we predicted the novel miRNAs through mapping the unassigned small RNA sequences to the genomes of all animals in UCSC database. Therefore, the novel miRNAs identified in this study are not previously characterized in any other species under the experimental and analytical condition at that time. Li et al. reported that novel miRNAs are lowly expressed in chicken skeletal muscle [55]. In this study, we also found that the read number of each novel miRNA was much lower than the identified miRNAs. Only two novel miRNAs (novel-mir-8 with 3795 reads at E13 and 5154 at E27 and novel-mir-14 with 831 reads at E13) had read counts greater than 100 in the libraries (Table 4). These results indicate that novel miRNAs are masked by highly expressed miRNAs because of the low abundance [56]. Targets prediction showed that the novel miRNA candidates corresponded to 3867, 2680 and 4239 target genes and 8362, 3847 and 13099 genomic loci, respectively (Table 5). For predicted targets of the novel miRNAs, we performed GO (gene ontology) and KEGG (Kyoto Encyclopedia of Genes and Genomes) pathway analysis. Among the pathways, the metabolic pathways contained the highest number of targets genes (344, 235, and 370 targets in the three libraries respectively) (Table S5), suggesting that metabolism is a major event at different development stages. In addition, many muscle development relevant pathways, such as MAPK signaling pathway, mTOR signaling pathway, Wnt signaling pathway and calcium signaling pathway were also discovered from the KEGG pathway analysis of the targets of novel miRNAs suggesting the novel miRNAs predicted in this study may play some roles in muscle development.

**Table 4:** Summary of novel miRNAs.

Novel miRNA name	Novel miRNA sequence	Cou-E13	Cou-E19	Cou-E27
**novel-mir-1**	CGCTCAGTAGTCAGTGTAGATTC	27	0	36
**novel-mir-10**	AGGTCCCTGTTTGGGCGCCA	6	0	0
**novel-mir-11**	CTGTTACTGTTCTTCTGATGG	8	0	17
**novel-mir-12**	CAGCCTACCGACTGTTGTTGCC	0	10	11
**novel-mir-13**	GGGGGCCGGGCCGGGCCGGCGG	0	36	12
**novel-mir-14**	TGAGAACTGAATTCCATGGACT	0	831	0
**novel-mir-15**	GGGACGGGGACGGGGACGGG	0	11	5
**novel-mir-16**	CGGCGGCGGCGGTGGCGGCGGCG	0	0	6
**novel-mir-17**	ATAGCTCTTTGAATGGTACTGC	0	0	8
**novel-mir-18**	TGGGACTTTGTAGGCCAGTTGA	0	0	5
**novel-mir-19**	TGTATTGGAACACTACAGCTC	0	0	8
**novel-mir-2**	GGCGCCGCCCGCCCCGCGCGC	15	0	0
**novel-mir-20**	ATATATGTGGTCAGACCTATC	0	0	12
**novel-mir-21**	TGAGGAACTCAGGCGGCTCGA	0	0	5
**novel-mir-22**	TTGGACTTACTGTGCATGTGCTA	0	0	6
**novel-mir-23**	GGGGATGTGTAAAAGAAGAAGCG	0	0	11
**novel-mir-3**	TGTATTGGAACACTACAGCTCC	7	0	0
**novel-mir-4**	GGGGGCCGGGCCGGGCCGGCGGG	100	0	0
**novel-mir-5**	AATGTGGAGTTGGCTGGGCTGG	9	0	7
**novel-mir-6**	TCACATTTGCCTGCAGAGATTT	15	6	21
**novel-mir-7**	CAGACCATTCTGGGCTGCCTCA	10	0	14
**novel-mir-8**	TGAGAACTGAATTCCATGGACTG	3795	0	5154
**novel-mir-9**	AGTTACATGTATGCATCGAGCA	16	0	19

**Table 5:** Summary of novel miRNAs target prediction.

Sample names	miRNAs number	Target number	Target loci number
**E13**	11	3867	8362
**E19**	5	2680	3847
**E27**	18	4293	13099

Notably, the expression of novel-mir-8 in E13 and E27 were higher than any other identified novel miRNAs and there was no expression at E19. On the contrary, novel-mir-14 was only highly expressed at E19, but was not expressed at E13 and E27. Considering the fact that the embryonic breast muscle growth of duck is fastest at E19 (Table 4), we speculated that novel-mir-8 could be a suppressor of the embryonic breast muscle growth of duck, while novel-mir-14 was a promoter.

To further explore the roles of novel-mir-8 and novel-mir-14 in the embryonic breast muscle growth of Pekin duck, we investigated the function of their targets using the DAVID database for annotation, visualization and integrated discovery (with default parameters) [57]. Because there is no genomic information available for duck, we annotated these targets against the chicken genome. Through the gene name batch viewer analysis, 547 and 878 GenBank_Accession of the targets for novel-mir-8 and novel-mir-14 were linked with gene names in GenBank (Table S6). The results of GO analysis showed that there were 124, 27 and 32 terms in the three main categories of biological process, cellular component and molecular function, respectively, for the targets of novel-mir-8 and 241, 34 and 33 terms for that of novel-mir-14, respectively (Table S7). In the KEGG pathway analysis for the targets of novel-mir-8, we found six significant pathways (*P<0.05*). Among the six pathways, MAPK signaling pathway was the pathways with the highest number of targets (18 targets) (Table S8). Mounting studies have shown that MAPK signaling pathway is involved in skeletal muscle development and modulation of skeletal muscle fiber type [58]–[62]. Three pathways including the adipocytokine signaling pathway, were found to be significant enriched (*P<0.05*) in the KEGG pathway analysis for the targets of novel-mir-14 (Table S9). Previous studies have shown that increased volume and number of adipocyte are positively correlated with leptin production [63], [64] and negatively correlated with production of adiponectin (also known as ACRP30), a hormone that decreases hepatic gluconeogenesis and increases lipid oxidation in muscle [65], [66]. Leptin signaling has also been implicated in the pathogenesis of arterial thrombosis [67]. Therefore, functional annotation of the novel miRNA targets suggest that novel-mir-8 and novel-mir-14 might play important roles in the embryonic breast muscle development of duck, which promoted us to validate the function of these two novel miRNAs and their targets.

### Validation of biological variability between samples of a stage

**Figure 5:**
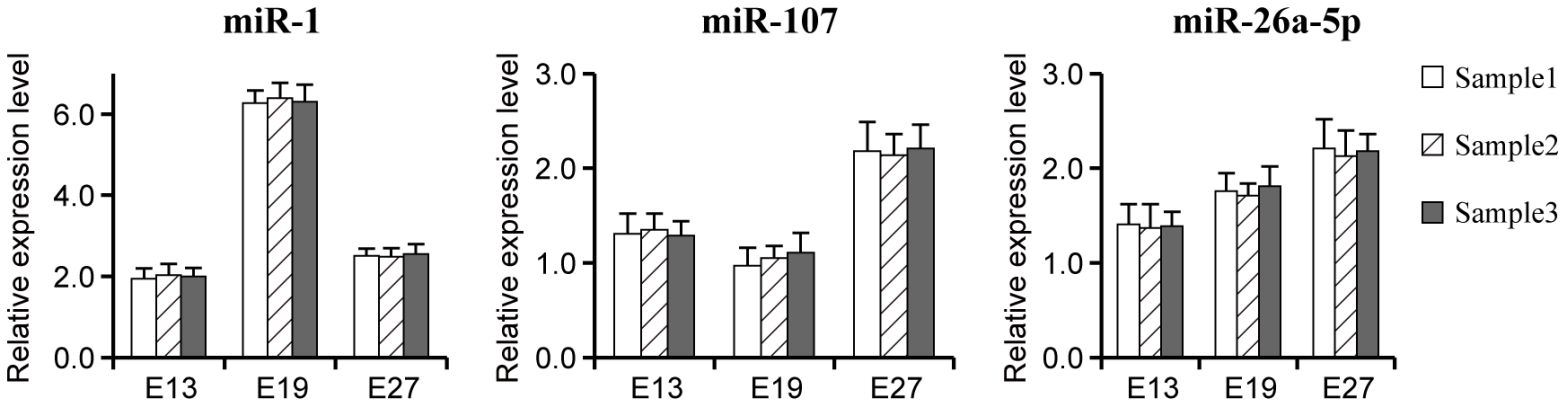
Validation of biological variability among samples of a stage. Note: BME11, BME13, BME16, BME19, BME23, BME27 refer to breast muscle at stage E11, E13, E16, E19, E23, E27 respectively, LM-Leg muscle, H-Heart, L-Liver, K- Kidney, MS-Muscular stomach, SI- Small intestine, AF-Abdominal fat, SF-Skin fat.

To minimize the effect of biological variability, three full-sib embryos were used to collect breast muscle samples at stage E13, E19 and E27. Then, we isolated total RNA from these three embryos of a stage and pooled them together to generate a RNA pool of this stage similar to the pooling for the high-throughput sequencing. Because the genetic variability in studies is extensive and inevitable, further validation of miRNAs expression changes need be performed between individuals of a specific stage. Therefore, we randomly selected three highly expressed miRNAs (miR-1, miR-107, and miR-26a-5p) to performed stem- loop qRT-PCR analysis in each sample (Fig. 5). The results showed that there were no significant differences among samples of a stage. This indicates that the effect of biological variability is not significant in this study and the data used in this study is reliable.

### Validation of identified miRNAs

**Figure 6:**
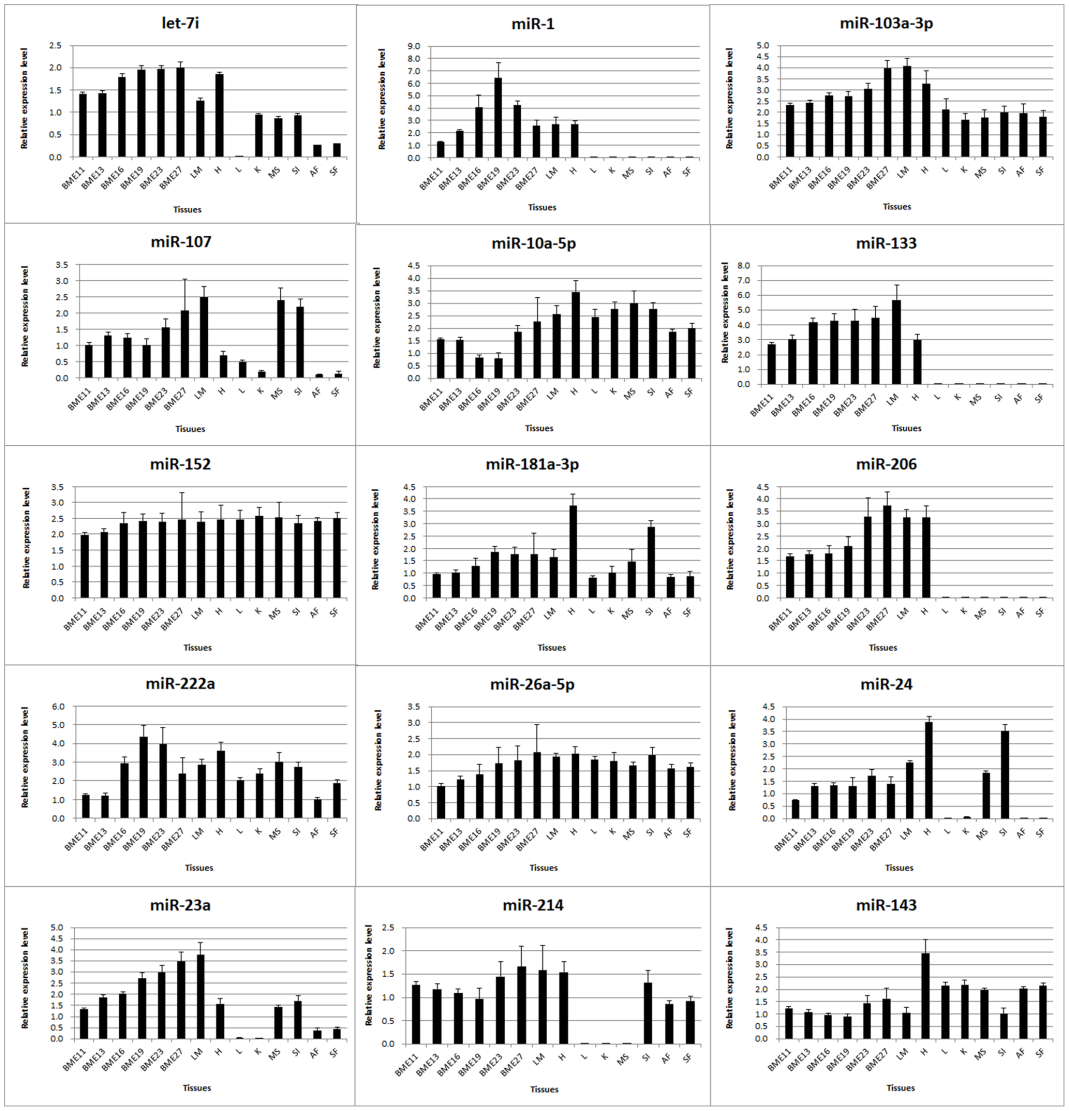
Validation of miRNA expression by qRT-PCR.

Muscle-specific miRNAs are predominantly expressed in muscle-related tissues or organs and are involved in a range of processes including myogenesis (proliferation, differentiation, and fiber type specification), muscle regeneration, hypertrophy, and dystrophy [13], [68]–[71]. Therefore, understanding the miRNAs expression pattern can reveal the potential function of the miRNAs. To validate the identified miRNAs in embryonic breast muscle of Pekin duck, stem-loop qRT-PCR analysis of 15 identified duck miRNAs was performed in different tissues or organs (leg muscle, heart, liver, kidney, muscle stomach, small intestine, abdominal fat, skin fat) at E27 and in breast muscle at various developmental stages (E11, E13, E16, E19, E23, E27). Among the 15 miRNAs, 14 miRNAs (93.3%) were in agreement with the expression pattern found in the high-throughput sequencing data (Fig. 6), indicating the high-throughput sequenced data and analysis methods are reliable. Through comparing the 15 miRNAs expression profiles among tissues, we found that the three muscle-specific miRNAs (miRNA-206, miRNA-1, and miRNA-133) were highly expressed exclusively in in muscle tissue or related organs (breast muscle, leg muscle, and heart), while six myogenesis-related miRNAs (miR-181a-3p, miR-103a-3p, miR-107, miR-10a-5p, miR-222a, and miR-26a-5p) and two highly expressed miRNAs (miR-152 and miR-143) could be detected in all tissues. Interestingly, the expression level of miRNA-152 was approximately equal in all tissues/organs. The remaining 4 miRNAs were not expressed in one or several tissues or organs, like let-7i which had no expression in liver, miRNA-23a were not express in liver and kidney, miRNA-24 hardly showed any expression in liver, kidney, abdominal fat and skin fat and miR-214 could not be detected in liver, kidney, and stomach. The expression of the 15 validated miRNAs were all highly expressed in muscle-related tissues (breast skeletal muscle, leg muscle, and heart) (Fig. 6) suggesting that these miRNAs might play some roles in skeletal muscles development.

To further explore the temporal expression of the 15 miRNAs validated above in the developing embryonic breast muscle of Pekin duck, we performed stem-loop qRT-PCR analysis of the miRNAs in embryonic breast muscle tissues at E11, E13, E16, E19, E23, and E27. The results showed that the analyzed miRNAs could be divided into three groups according to their expression profiling. The expression profiling of miRNAs in group 1 (miR-1, miR-181a-3p, and miR-222a) peaked at E19. Conversely, the miRNAs expression patterns in group 2 (miR-107, miR-10a-5p, miR-214, and miR-143) were lowest at E19. Given that E19 is the fastest growth stage of embryonic breast muscle of Pekin duck according to our prior research, the miRNAs in group 1 might be the potential promoters and the miRNAs in group 2 might be the potential inhibitors for embryonic breast muscle development of duck. The expression of miRNAs in group 3 (let-7i, miR-103a-3p, miR-133, miR-206, miR-26a-5p, miR-24, and miR-23a) increased in the embryonic breast muscles from E11 to E27 (Fig. 6). These results suggested that the miRNAs in group 3 may be involved in the growth and functional maintenance of embryonic breast muscles of duck.

### Validation of novel-mir-8 and novel-mir-14 and their targets

**Figure 7:**
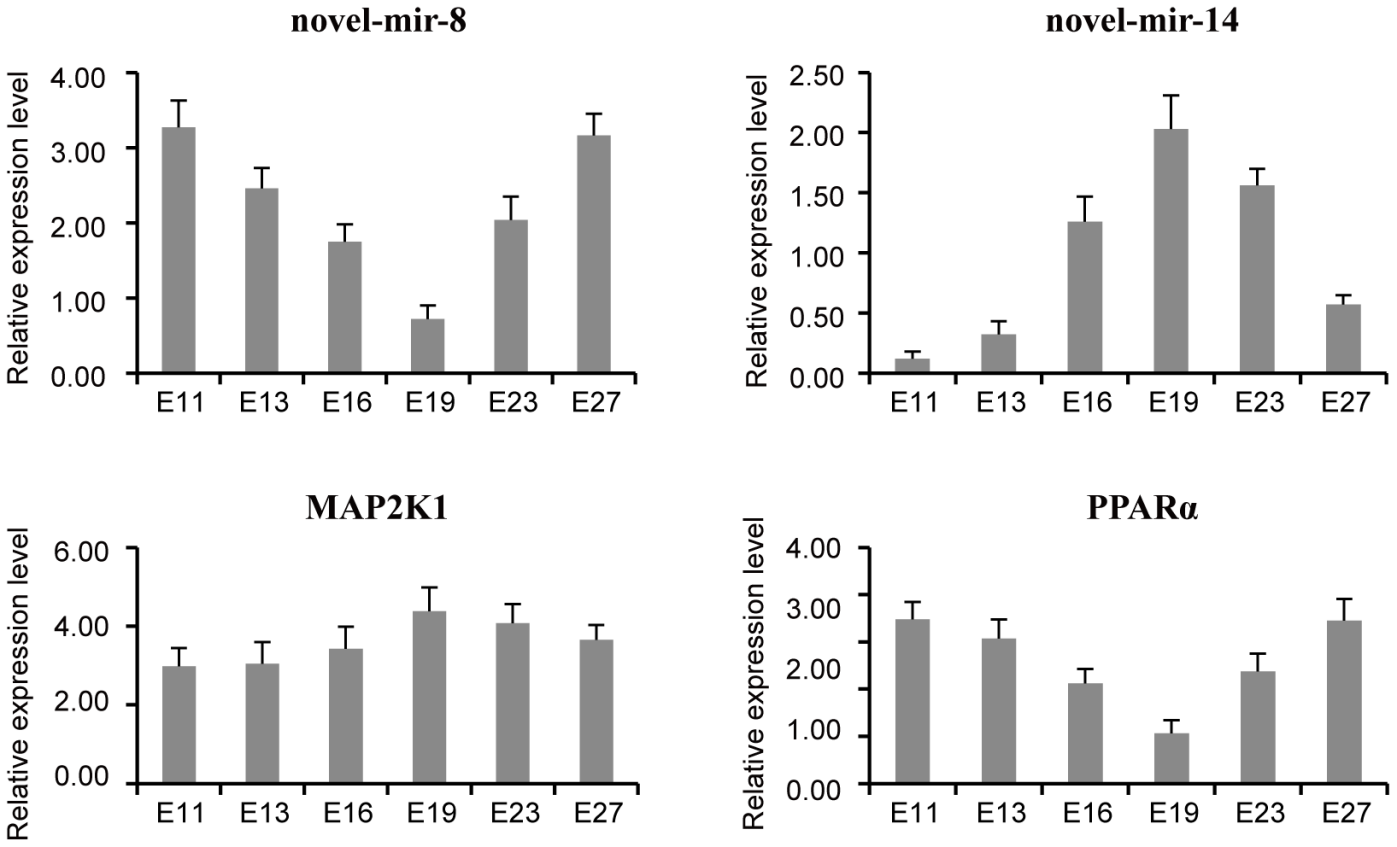
Validation of novel-mir-8 and novel-mir-14 and their targets.

Because of the high expression of novel-mir-8 and novel-mir-14 in prenatal breast muscle of duck at some specific stages (novel-mir-8 is highly expressed at E13 and E27, novel-mir-14 is highly expressed at E19) and the pathways of muscle development or fat deposition involved in by their targets, we were urged to carry out the expression validation of the two novel miRNAs and some of their targets by stem-loop qRT-PCR. We carried out expression profiles analysis of novel-mir-8, novel-mir-14, mitogen-activated protein kinase kinase 1 (MAP2K1, one of the target of novel-mir-8 in MAPK signaling pathway) and peroxisome proliferator-activated receptor alpha (PPARα, one of the target of novel-mir-14 in adipocytokine signaling pathway) in embryonic breast muscle of Pekin duck at E11, E13, E16, E19, E23 and E27. The results showed that the expression of novel-mir-8 and PPARα simultaneously reached minimal expression level at E19. In contrast, the expression of novel-mir-14 and MAP2K1 reached maximal expression at E19 (Fig. 7). Evidence showed that activated MAP2K1, also known as MEK1, is a positive regulator in the mid-stage of skeletal myogenesis [72] and that peroxisome proliferator-activated receptor alpha (PPARα), which enhances peroxisomal β-oxidation [73] and activation of lipoprotein lipase [74], can promote fat deposition. Based on the expression profiles of novel-mir-8, novel-mir-14, MAP2K1 and PPARα as well as the roles of MAP2K1 and PPARα as reported previously, we speculated that novel-mir-8 and novel-mir-14 might be the promoter and inhibitor, respectively, for the embryonic breast muscle development in Pekin duck. However, further follow up studies, such as RNAi, are needed to verify this hypothesis.

## Materials and Methods

### Ethics statement

All samples' collection and subsequent experiments were approved by the Ethical and Animal Welfare Committee of Beijing, China. Birds involving slaughtering in this study were humanely sacrificed as necessary to ameliorate their suffering.

### Tissue collection, small RNA isolation and cDNA libraries construction

Three full-sib embryos of Pekin duck at stage E11, E13, E16, E19, E22 and E27 were collected into sterile physiological saline immediately after removal from the hatching eggs. Pekin duck tissue samples used for stem-loop real-time reverse transcription polymerase chain reaction (qRT-PCR) analysis included leg muscle, heart, liver, kidney, intestines, abdominal fat, and skin fat from stage E27 and breast muscles from stage E11, E13, E16, E19, E22 and E27. These tissues were snap-frozen in liquid nitrogen and stored at −80°C. Total RNA was isolated using TRIzol reagent (Invitrogen) from all the tissue samples. For stage E13, E19, and E27, the isolated total RNA was pooled and used for the generation of the small RNA libraries where the population of recovered small RNAs, ranging in size from 18 to 30 nucleotides, was purified using 15% polyacrylamide gel. Then, 5′ adaptors (Illumina, USA) were ligated to the purified small RNAs followed by purification of ligation products on Novex 15% TBE-urea gel. The 5′ ligation products were then ligated to 3′ adaptors (Illumina) and products with 5′ and 3′ adaptors were purified using Novex10% TBE-urea gel (Invitrogen). Subsequently, reverse transcription reactions were performed using the RT primer, and PCR reactions were performed using the forward and reverse Illumina primers. The PCR product was purified via phenol/chloroform extraction and ethanol precipitation and was delivered to the Beijing Genomics Institute (BGI) (Shenzhen, China) for sequencing on an Illumina Gnome Analyzer.

### Small RNA analysis

The adaptor/acceptor sequences and low quality reads were removed and the contaminated reads formed by the adaptor-adaptor ligation was cleaned by a software developed by BGI, hereby the high-quality reads were obtained. Because there is no genomic information of duck available, we compared the high-quality reads with GenBank noncoding RNA database (http://www.ncbi.nlm.nih.gov/) and Rfam database (http://www.sanger.ac.uk/software/Rfam). Sequences with highest similarity to rRNA, tRNA and sncRNA were removed. Subsequently, the high-quality reads were compared with chicken miRNAs, and with other animal miRNAs in miRBase 19.0. Finally, reads number of each miRNA candidates was counted and compared between different tissues. In view of the miRNA diversities in different species, two nucleotide mismatches were allowed in these analysis. The remaining unmatched small RNA fragments were blasted against the chicken genome sequence to identify the exon, intron and repeat sequences using SOAP 2.0 program [28].

### Prediction of novel miRNAs

To discover potential novel miRNAs precursor sequences, unique sequences that have more than 10 hits to the chicken genome or match to known non-coding RNAs were removed. Then the flanking sequences (150 nt upstream and downstream) of each unique sequence were extracted for secondary structure analysis with Mfold [http://www.bioinfo.rpi.edu/applications/mfold] and then evaluated by Mireap [http://sourceforge.net/projects/mireap/]. After prediction, the potential miRNA loci were examined carefully based on the distribution and numbers of small RNAs on the entire precursor regions. Those sequences residing in the stem region of the stem-loop structure and ranging between 20–22 nt with free energy hybridization lower than −18 kcal/mol were considered to be potential novel miRNA candidates [75].

### Prediction of novel miRNAS and functional annotation of the target genes

RNAhybrid (http://bibiserv.techfak.uni-bielefeld.de/rnahybrid) [76] was used to predict the targets of novel miRNAs, complying with the following criteria in seed region: (1) No mismatch between 1–9 nt on the 5′ end; (2) G-U was permitted, but the number can't exceeds three. The next thing is DAVID (http://david.abcc.ncifcrf.gov/) [57] being used for the functional annotation of the predicted targets. Because there is no genomic information available for duck, we annotated tgohese targets against the chicken genome using the GenBank Accession of the targets of novel miRNAs.

### miRNA expression analysis

Comparison of the identified or novel miRNA expression between two samples was conducted to identify those differentially expressed miRNAs. The expression of miRNAs was shown in two samples by plotting a Log2-ratio figure and a Scatter Plot. The procedures were as follows: (1) Normalize the expression of miRNAs in two samples (E19 and E13 or E19 and E27) to get the expression of transcript per million (TPM). Normalized expression (NE) = Actual miRNA count/Total count of high-quality or novel reads. (2) Calculate fold-change and P-value from the normalized expression. Then generate the Log2-ratio plot and scatter plot. Fold-change formula: Fold change = log2 (E13/E19 or E27/E19). P-value formula:
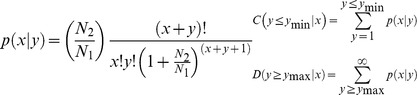



The x and y represented normalized expression levels, and the N_1_ and N_2_ represented total count of a given miRNA in the small RNA libraries of E19 and E13 or E19 and E27, respectively.

Some members of differentially expressed miRNAs were validated using stem-loop qRT-PCR with SYBR Green [77]. The primers for stem-loop qRT-PCR are listed in Table 6. qRT-PCR was carried out with an iCycler IQ5 Multicolor Real-Time PCR Detection System (Bio-Rad, USA) and SYBR Green PCR Master Mix (TaKaRa, Dalian, China) in a 20 µl reaction. The reaction mixtures were incubated in a 96-well plate at 95°C for 30 s, followed by 40 cycles of 95°C for 10 s, 60°C for 10 s, and 68°C for 20 s. All reactions were run in triplicate. The threshold cycle (Ct) was defined as the cycle number at which the fluorescence intensity passed a predetermined threshold. The quantification of each miRNA relative to U6 gene was calculated using the equation: N = 2^−ΔΔCt^.

**Table 6:** Primers used in this study.

miR-name	Primer sequences (5′–3′)
**let-7i forward**	GCGCGTGAGGTAGTAGTT
**miR-1 forward**	GCGCGCTGGAATGTAAAGAAG
**miR-103a-3p forward**	CGAGCAGCATTGTACAGGGCTATG
**miR-107 forward**	ACACTCCAGCTGGGAGCAGCATTG
**miR-10a-5p forward**	CCTGTAGATCCGAATTTGTG
**miR-133 forward**	GCGCGCTTTGGTCCCCTTCAA
**miR-152 forward**	GGTCAGTGCATGACAGAAC
**miR-181a-3p forward**	AACATTCAACGCTGTCGGT
**miR-206 forward**	GCGCGCTGGAATGTAAGGAAG
**miR-222a forward**	CGAGCTACATCTGGCTACTGGGTC
**miR-26a-5p forward**	GGCTTCAAGTAATCCAGGATAGGC
**miR-24 forward**	TGGCTCAGTTCAGCAGGA
**miR-23a forward**	ATCACATTGCCAGGGATTTC
**miR-214 forward**	TACAGCAGGCACAGACAGG
**miR-143 forward**	CCTGAGATGAAGCACTGTAGC
**novel-mir-8 forward**	GGTGAGAACTGAATTCCATGGACTG
**novel-mir-14 forward**	GGGTGAGAACTGAATTCCATGGAC
**miRNA reverse**	CTCAACTGGTGTCGTGGA
**U6-forward**	CTCGCTTCGGCAGCACA
**U6-reverse**	AACGCTTCACGAATTTGCGT
***MAP2K1-*** ** forward**	CGGAAAGACTACAGGGAAC
***MAP2K1-*** ** reverse**	AGTCAGGAGGAGGAATCG
***PPARα-*** ** forward**	CGCTGCCATCATTTGCTG
***PPARα-*** ** reverse**	AAGTTGTCGGAGGTCAGCC
***β-actin-*** ** forward**	GCTATGTCGCCCTGGATTTC
***β-actin-*** ** reverse**	CACAGGACTCCATACCCAAGAA

### The qRT-PCR analysis of the target genes of novel-mir-8 and novel-mir-14

The SYBR PrimeScript RT-PCR Kit (TaKaRa, Dalian, China) and a reference gene (*β-actin*) were used for detecting the expression of MAP2K1 (a of the target of novel-mir-8) and PPARα (a of the target of novel-mir-14). The qRT-PCR reactions were carried out with an iCycler IQ5 Multicolor Real-Time PCR Detection System (Bio-Rad, USA). The qRT-PCR reaction contained 1 µL of cDNA template, 12.5 µL of SYBR Premix ExTaq, 9.5 µL of sterile water, and 1 µL of each gene-specific primer (Table 6). Thermal cycling parameters were 1 cycle at 95°C for 2 min, 40 cycles of 95°C for 15 s, 60°C for 34 s. Dissociation curve analysis was done after each real time reaction to ensure that there was only one product. qRT-PCR analysis of each sample was repeated for three times. The quantification of each gene relative to *β-actin* gene was calculated using the equation: N = 2^−ΔΔCt^.

### Data deposition

Data described in this study is available in the NIH Short Read Archive (SRA) under accession number PRJNA098308.

## Supporting Information

Table S1 **Mapping statistics of samples studied in this study.**
(DOCX)

Table S2 **miRNA nucleotide bias at each position.**
(XLSX)

Table S3 **Expression abundance of identified miRNAs in E13, E19, and E27 libraries.**
(XLSX)

Table S4 **Summary of differentially expressed identified miRNAs.**
(XLS)

Table S5 **The pathway annotation of the targets of novel miRNAs in E13, E19 and E27.**
(XLSX)

Table S6 **The gene name batch viewer analysis of the targets of novel-mir-8 and novel-mir-14.**
(XLSX)

Table S7 **The GO results of the targets of novel-mir-8 and novel-mir-14.**
(XLSX)

Table S8 **The pathways and the relative genes among the targets of novel-mir-8.**
(XLSX)

Table S9 **The pathways and the relative genes among the targets of novel-mir-14.**
(XLSX)
