# Supplementary material for: Identification and Profiling of MicroRNAs in the Embryonic Breast Muscle of Pekin Duck
Source: PLoS One. 2014 Jan 23;9(1):e86150. doi: 10.1371/journal.pone.0086150 (PMC3900480; doi:10.1371/journal.pone.0086150)
Supplement: Table S1 — Mapping statistics of samples studied in this study. (DOCX) [file pone.0086150.s001.docx]

**Table S1. Mapping statistics of samples studied in this study**

| samples | Items | Unique sRNA | Total sRNA |
| --- | --- | --- | --- |
| E13 | Total sRNA | 822873（100%） | 14580115（100%） |
|  | Mapping to genome | 107578（13.07%） | 10273938（70.47%） |
| E19 | Total sRNA | 389934（100%） | 13016970（100%） |
|  | Mapping to genome | 63253（16.22%） | 11171826（85.83%） |
| E27 | Total sRNA | 508157（100%） | 15549081（100%） |
|  | Mapping to genome | 77459（15.24%） | 13010462（83.67%） |
